# Internalization of Foldamer-Based DNA Mimics through a Site-Specific Antibody Conjugate to Target HER2-Positive Cancer Cells

**DOI:** 10.3390/ph14070624

**Published:** 2021-06-28

**Authors:** Valentina Corvaglia, Imène Ait Mohamed Amar, Véronique Garambois, Stéphanie Letast, Aurélie Garcin, Céline Gongora, Maguy Del Rio, Caroline Denevault-Sabourin, Nicolas Joubert, Ivan Huc, Philippe Pourquier

**Affiliations:** 1 Center for Integrated Protein Science, Department of Pharmacy, Ludwig-Maximilians-Universität, 81377 Munich, Germany; valentina.corvaglia@cup.lmu.de (V.C.); ivan.huc@lmu.de (I.H.); 2 GICC EA7501, Equipe IMT, Université de Tours, 10 Boulevard Tonnellé, F-37032 Tours, France; imene.aitmohamedamar@etu.univ-tours.fr (I.A.M.A.); stephanie.letast@univ-tours.fr (S.L.); caroline.denevault@univ-tours.fr (C.D.-S.); nicolas.joubert@univ-tours.fr (N.J.); 3 Institut de Recherche en Cancérologie de Montpellier, INSERM U1194, Université de Montpellier, F-34298 Montpellier, France; veronique.garambois@icm.unicancer.fr (V.G.); aurelie.garcin@inserm.fr (A.G.); celine.gongora@inserm.fr (C.G.); maguy.delrio@icm.unicancer.fr (M.D.R.)

**Keywords:** DNA mimics, foldamer, antibody-drug conjugate, trastuzumab, HER2

## Abstract

Inhibition of protein–DNA interactions represents an attractive strategy to modulate essential cellular functions. We reported the synthesis of unique oligoamide-based foldamers that adopt single helical conformations and mimic the negatively charged phosphate moieties of B-DNA. These mimics alter the activity of DNA interacting enzymes used as targets for cancer treatment, such as DNA topoisomerase I, and they are cytotoxic only in the presence of a transfection agent. The aim of our study was to improve internalization and selective delivery of these highly charged molecules to cancer cells. For this purpose, we synthesized an antibody-drug conjugate (ADC) using a DNA mimic as a payload to specifically target cancer cells overexpressing HER2. We report the bioconjugation of a 16-mer DNA mimic with trastuzumab and its functional validation in breast and ovarian cancer cells expressing various levels of HER2. Binding of the ADC to HER2 increased with the expression of the receptor. The ADC was internalized into cells and was more efficient than trastuzumab at inhibiting their growth in vitro. These results provide proof of concept that it is possible to site-specifically graft high molecular weight payloads such as DNA mimics onto monoclonal antibodies to improve their selective internalization and delivery in cancer cells.

## 1. Introduction

Nucleic acids (NAs) are key players in the regulation of most cellular processes. This role relies on both the ability of two complementary NA strands to hybridize through Watson–Crick purine/pyrimidine base-pairing, and on NA interactions with various proteins via specific features of their surfaces, including shape, charge distribution or groove width, as defined by the base sequence. Thus, disruption of NA base pairing or NA-protein interactions may have drastic consequences on cell fate, justifying high interest for synthetic NA mimics that could alter such processes for diagnostic purposes or therapeutic applications. Peptide nucleic acids [1] (PNAs) and locked nucleic acids [2] (LNAs) represent successful examples of DNA mimic development to modulate base pairing and control gene expression in various biological contexts [3,4]. Regarding the inhibition of nucleic acid-protein interactions, several reports showed that it can be achieved with small molecules such as DNA ligands [5,6,7], ‘interfacial’ inhibitors targeting specific DNA-protein complexes [8,9] or with oligodeoxynucleotide decoys targeting specific transcription factors [10,11,12]. The de novo design and synthesis of molecules (e.g., peptides) that mimic DNA surface features is another attractive strategy to interfere with DNA-protein interactions [13]. It is inspired by the description of more than a dozen of naturally occurring DNA mimic proteins that impair the biological functions of prokaryotic or eukaryotic enzymes involved in various cellular processes, presumably via a competitive inhibition of their interaction with NAs [14]. Recently, we have reported the synthesis of unique oligoamide-based foldamers that adopt single helical conformations and mimic the array of negatively charged phosphate moieties in double-stranded B-DNA. We found that these DNA mimics could alter the function of DNA interacting enzymes, such as Topoisomerase I and HIV-1 integrase in vitro [15]. We further demonstrated that, depending on the spatial distribution and the nature of anionic side chains on the foldamers, it was possible to inhibit the activity of DNA-interacting enzymes in a selective manner [16]. We also showed that these foldamers could inhibit the growth of cancer cells. However, this effect could only be observed when delivery was carried out by a transfection agent [15]. Indeed, the polyanionic nature of the DNA mimics and their poor lipophilicity—in other words their resemblance to DNA—precluded their entry into cells, pointing towards the need for derivatives with enhanced cell penetration ability. Another issue resides in the possibility to selectively target cancer cells as DNA mimics can also inhibit the growth of normal cells (unpublished observations). These data led us to consider several vectorization options of DNA mimics, including the development of an antibody-drug conjugate (ADC). ADCs result from the grafting of several molecules (e.g., cytotoxic agents) onto a monoclonal antibody targeting a specific internalizing antigen that is overexpressed at the surface of cancer cells. The design, synthesis and development of ADCs have made substantial progress within the past ten years [17]. Original payloads exhibiting new mechanisms of action, site-specific bioconjugation technologies, improved linkers [18,19] allowing for a better control of the drug-to-antibody ratio (DAR), optimization of ADC internalization and payload release [20,21] led to second and third generation ADCs with a more effective therapeutic index. There are currently ten ADCs clinically approved by the FDA for the treatment of hematological malignancies and solid tumors, and a growing number of other molecules are either in preclinical studies or in early phase clinical trials [17,22].

The objective of our study was to synthesize an ADC using a DNA mimic foldamer as a new payload in order to specifically target cancer cells and facilitate its internalization and delivery into cells in the absence of transfecting agents. Here, we report the bioconjugation of a 16-mer DNA mimic with the HER2-specific monoclonal antibody trastuzumab, and present experimental evidences of the biological activity of this ADC in several breast and ovarian cancer cell lines expressing various levels of HER2. We found that binding of the ADC to HER2 increased with the expression of the receptor. We also showed that the ADC was internalized into cells and was more efficient than trastuzumab at inhibiting their growth in vitro. Together, these data provide a proof of concept that it is possible to graft high molecular weight payloads such as DNA mimics onto monoclonal antibodies with a controlled drug-to-antibody ratio (DAR) that is compatible with other ADCs currently used in the clinic. Our results further extend the potential of using the ADC strategy to deliver synthetic payloads that are not highly cytotoxic drugs, but that would instead be used to potentiate the action of conventional chemotherapies.

## 2. Results and Discussion

In order to implement cell delivery of the DNA mimic foldamers, several targeting strategies were considered, including the synthesis of an ADC. This class of therapeutic agents where a cytotoxic drug is grafted onto a monoclonal antibody with a fixed DAR has emerged in the last decade and is rapidly expanding with the recent approval of several molecules [17,20]. Trastuzumab is a monoclonal antibody targeting HER2 that is approved for the treatment of HER2-positive breast cancers. After the binding of trastuzumab on its HER2 specific antigen, the trastuzumab-HER2 complex is able to internalize efficiently. Therefore, trastuzumab has been conjugated to highly cytotoxic drugs such as the tubulin polymerization inhibitor emtansine or the DNA topoisomerase I poison deruxtecan, leading respectively to the two ADCs, ado-trastuzumab emtansine (T-DM1 or Kadcyla^®^) and fam-trastuzumab deruxtecan-nxki (DS-8201a or Enhertu^®^), that are approved by the FDA against HER2-positive breast cancer [17]. A third ADC, vic-tratuzumab duocarmazine (SYD985), using the alkylating agent duocarmycin as a payload, was also developed [23] and is currently in phase 3 trial.

**Figure 1:**
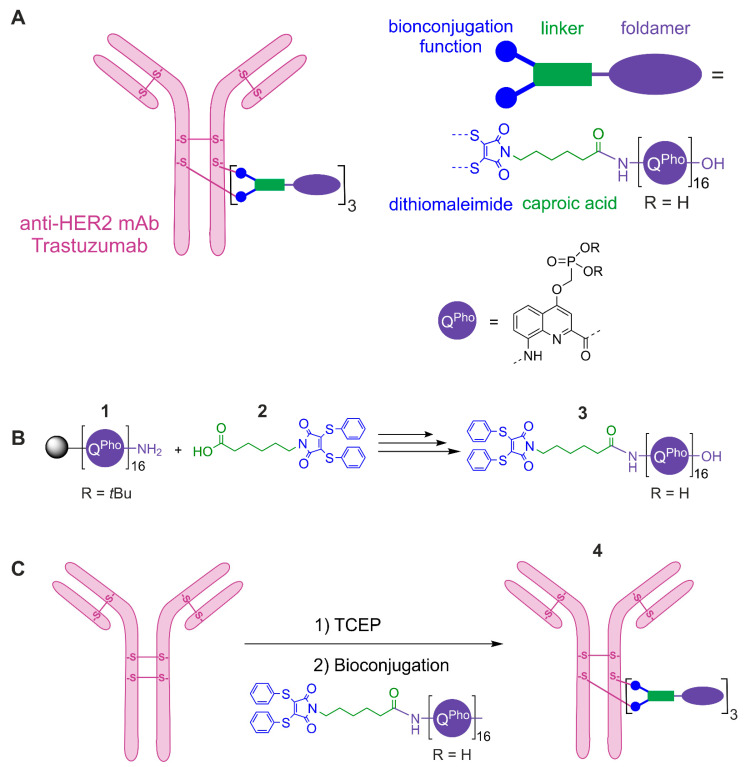
(**A**) Schematic representation of a site-specific ADC comprising the anti-HER2 trastuzumab conjugated to a polyanionic foldamer via a diphenylthio-maleimido-caproic acid linker (diphenyltio-Mal-Cap) with a DAR of 3 (drug-to-antibody ratio, corresponding to the number of foldamers grafted onto the mAb). (**B**) Coupling reaction performed on solid phase between the protected foldamer **1** and the diphenylthio-Mal-Cap linker **2** to produce the diphenylthio-Mal-Cap-foldamer polyanionic **3** after resin cleavage and side chain deprotection. (**C**) The ADC **4** was obtained by complete reduction (TCEP) of all four interchain disulfide bridges followed by a site-specific bioconjugation reaction via a diphenylthio-Mal-Cap linker **2**. During the foldamer solid phase synthesis and the coupling reaction shown in (**B**), the phosphonate groups of the quinoline monomers are protected as *tert*-butyl esters. TCEP = tris(2-carboxyethyl)phosphine.

Based on this background, we selected trastuzumab to graft a 16-mer DNA mimic foldamer composed of 8-amino-2-quinolinecarboxylic acid monomers each bearing a negatively charged phosphonate residue in position 4 (Figure 1A) to produce an ADC able to specifically target the surface of HER2-positive cancer cells and then internalize to deliver its payload.

To achieve this goal, we designed a site-specific ADC **4** (Figure 1C) using a bifunctional non-cleavable linker. Considering the size of the payload and its potential mechanism of action [15], we decided to use a non-cleavable linker to graft payload **3** (Figure 1B) as the most straightforward strategy. Indeed, after internalization of the ADC, trastuzumab will be completely digested in the lysosome. Since the foldamer resists degradation by both nucleases and proteases [15], the generated metabolite will be very close in structure to the precursor **3** [17,24]. This approach thus does not compromise the foldamer’s mechanism of action. The selected linker included a diphenylthiomaleimide bioconjugation function as a next generation maleimide, able to rebridge previously reduced interchain disulfide bridges of an antibody while incorporating up to four desired payloads for an IgG1 [25,26,27,28]. The diphenylthio-maleimidocaproic acid **2** (diphenylthio-Mal-Cap, Figure 1B) was synthesized according to previously reported procedures [28]. We then prepared the protected 16-mer **1** (Figure 1B) on a low loading Wang resin via acid chloride activation of the monomers under neutral conditions using the Ghosez reagent (1-chloro-*N*,*N*,2-trimethylpropenylamine) [29], following microwave assisted foldamer solid phase synthesis (SPS) procedures [30,31,32].

The *N*-terminal functionalization of the foldamer with the diphenylthio-Mal-Cap linker **2** was performed on solid phase by activating in situ the caproic acid moiety of the linker as an acid chloride using triphenyl-phoshine, trichloroacetonitrile and collidine as a base [33]. Removal from the resin with concomitant cleavage of the *tert*-butyl phosphonate ester groups was performed by treatment with 95:2.5:2.5 TFA/H_2_O/*i*Pr_3_SiH (vol/vol/vol). The crude material was analyzed and purified by RP-HPLC. The pure product was characterized by ^1^H NMR in H_2_O/D_2_O and mass spectrometry (Figures S1–S3). Precursor **3** was then conjugated onto trastuzumab. Trastuzumab in BBS buffer was first incubated with a solution of TCEP in BBS under argon at 37 °C for 75 min to allow complete reduction of the interchain disulfide bridges. Then, a solution of diphenylthio-Mal-Cap-foldamer **3** in water was added and the reaction mixture was incubated for 2 h at 4 °C under stirring. Repeated ultrafiltration and filtration yielded the purified ADC **4** with an average DAR of 3.0 (for MS analysis and DAR calculation: see Table S1 and Figure S5).

The successful conjugation of the DNA mimic onto trastuzumab demonstrated that it is possible to graft high molecular weight payloads to therapeutic antibodies. Previously, NAs conjugation onto antibodies (IgG or fragments) has been described for siRNA, allowing a rapid clearance during plasma circulation and a specific uptake of siRNA in cancer cells [34,35]. Conversely to classical hydrophobic payloads, both siRNA and foldamers have the advantage of exhibiting hydrophilic properties, allowing better solubility in water and a bioconjugation process free of organic solvent.

**Figure 2:**
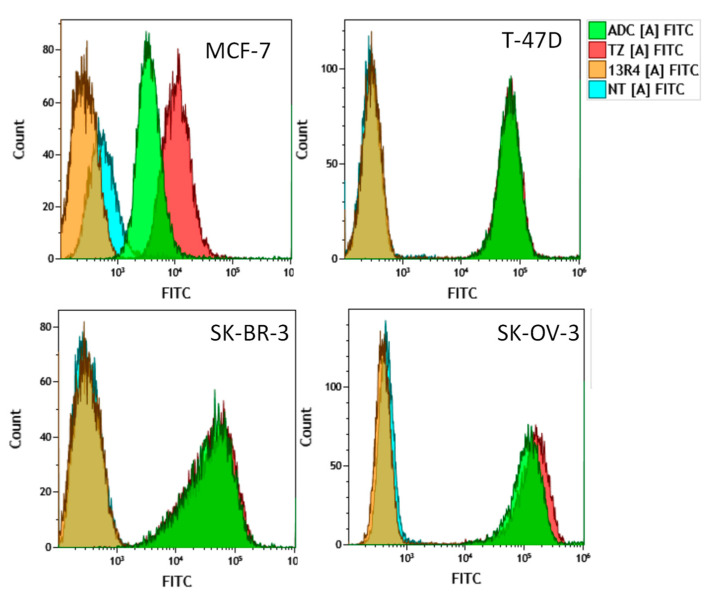
Binding of the trastuzumab-DNA mimic conjugate to breast (MCF-7, T-47D, SK-BR-3) and ovarian (SK-OV-3) cancer cells expressing various levels of HER2 was evaluated by FACS. Cells were incubated with 10 µg/mL of the trastuzumab-DNA mimic conjugate (ADC), trastuzumab (TZ) or the irrelevant 13R4 antibody for 1 h. Then, cells were washed with cold PBS, trypsinized and 1 × 10^6^ cells were analyzed by FACS. Untreated cells were used as a control.

We then characterized the biological activity of the newly synthesized ADC using three breast (MCF-7, T-47D and SK-BR-3) and one ovarian (SK-OV-3) cancer cell lines expressing various levels of HER2 [36,37,38,39]. We first wanted to validate whether the ADC could recognize its target at the cell surface, i.e., to assess whether the large payload (MW 4907 Da) alters in any way the HER2-trastuzumab interaction. We thus evaluated ADC binding to the receptor by FACS (Figure 2).

Binding of the ADC was compared to that of trastuzumab alone or of the irrelevant antibody 13R4 used as a negative control. While no binding could be evidenced for the 13R4 antibody with a mean fluorescence intensity value ranging from 0.3 to 0.6 (Table 1), a significant binding could be observed for the ADC in all the cell lines tested.

This binding increased with HER2 expression as mean fluorescence intensity varied from 14.2 in MCF-7 cells expressing very low levels of HER2, to ~84 in T-47D and SK-BR-3 cells expressing intermediate levels of HER2 and ~180 in SK-OV-3 cells expressing very high level of the receptor, respectively (Figure 2, Table 1). Interestingly, similar results were observed for trastuzumab alone, indicating that grafting of the DNA mimic with the caproic linker did not affect the binding capacity of the immunoconjugate.

**Figure 3:**
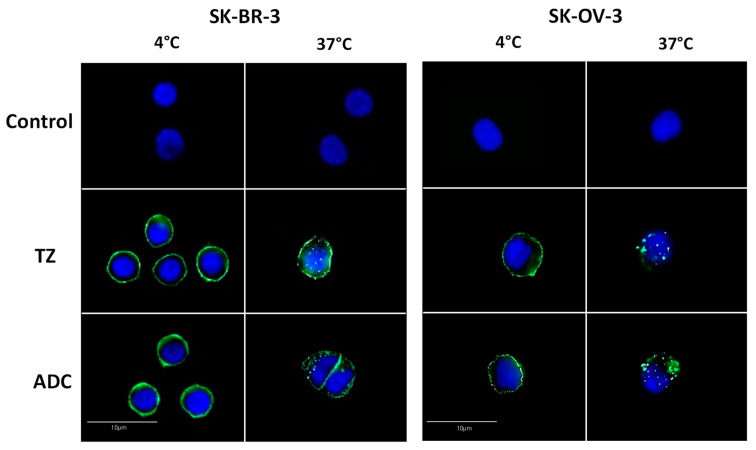
Internalization of the trastuzumab-DNA mimic conjugate in HER2 cells expressing HER2 at 4 °C and 37 °C as evaluated by immunofluorescence. Exponentially growing cells were incubated with 15 µg/mL of trastuzumab (TZ) or the trastuzumab-DNA mimic conjugate (ADC) for 2 h at 4 °C or 37 °C and were fixed. Localization of the antibodies was revealed by immunofluorescence using an FITC labeled antibody as described in the Materials and Methods section. Untreated cells were used as a negative control.

We then investigated whether the ADC could be internalized into cells. For this purpose, cells were treated with a fixed concentration of trastuzumab or of the ADC (15 µg/mL) for 2 h at 4 °C or at 37 °C, and immunofluorescence analyses were performed (Figure 3 and Figure S4).

The results of Figure 3 showed that, in SK-BR-3 and SK-OV-3 cells expressing the highest membrane levels of HER2, fluorescence was strictly localized at the cell surface whereas small intracellular patches of fluorescence could be detected when cells were treated at 37°C, which indicated an internalization of the ADC. Similar results were obtained for trastuzumab used as a positive control (Figure S4), in accordance with previous studies [40]. Internalization of both the ADC and trastuzumab was also observed in T-47D cells with a comparable change in fluorescence patches distribution between 4 °C and 37 °C, however, could not be evidenced in MCF-7 cells due to the very low level of expression of HER2 (Figure S4).

**Figure 4:**
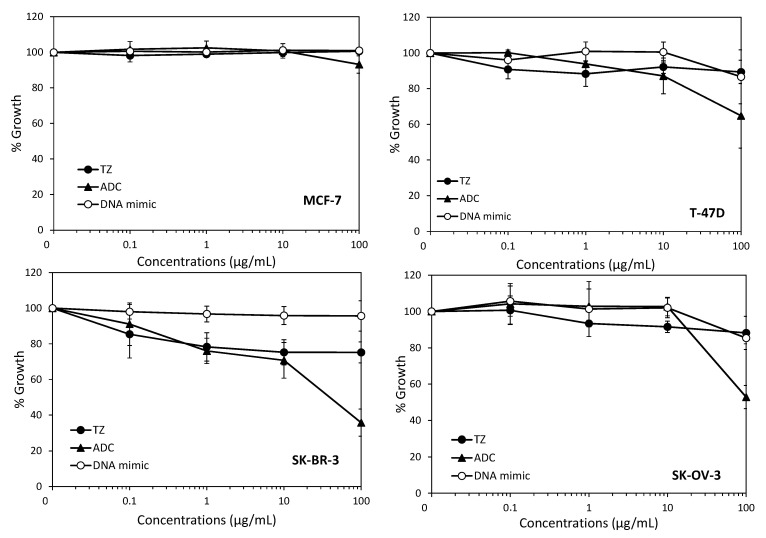
Effects of the trastuzumab-DNA mimic conjugate on cell growth. Cells were incubated with indicated concentrations of the trastuzumab-DNA mimic **4** (ADC), trastuzumab (TZ), or equivalent concentrations of the DNA mimic corresponding to a DAR 3 (DNA mimic) for 72 h, and cell growth was evaluated using the sulforhodamine B assay as described in Materials and Methods. Results are expressed as percentages of cell growth relative to untreated cells and are the mean ± sd of three independent experiments.

We then evaluated the effect of the ADC on the growth of breast and ovarian cancer cells using the standard sulforhodamine B in vitro assay (Figure 4). This effect was compared to the effect of equimolar concentrations of trastuzumab or of the (Q^pho^)_16_ DNA mimic alone, taking into account that the drug-to-antibody ratio was approximately three. The results showed that the DNA mimic alone did not have any effect on cell growth, which is in accordance with our previous observations [15] and confirms the difficulty of delivering these polyanionic moieties into cells without the use of a transfection agent. They also suggested that DNA mimics cannot exert their cytotoxic effect via an interaction with the cell surface. We found that, in our conditions, trastuzumab had only a minor effect on cell growth, even at the highest concentration used (100 µg/mL), with less than 20% growth inhibition, which is consistent with a previous study reporting the in vitro effects of this monoclonal antibody in the same models [41]. When compared to trastuzumab, our conjugate was more efficient at inhibiting cell growth only for the highest concentration of the drug used, i.e., 100 µg/mL (Figure 4). IC_50_s of our conjugate could only be obtained in SK-OV-3 and SK-BR-3 cells overexpressing HER2 (~50 µg/mL and ~100 µg/mL, respectively). Of note, 100 µg/mL of the trastuzumab-DNA mimic conjugate corresponds to a foldamer concentration of approximately 1.8 µM, which is in the same range of the concentrations that were transfected to obtain the maximal growth inhibition in HCT116 and HEK293 human cancer cell lines [15]. Other trastuzumab-drug conjugates were synthesized using highly cytotoxic agents as payloads, and were shown to be much more potent than trastuzumab in vitro and in vivo, even in trastuzumab-resistant models [41]. Such a higher potency was attributed to the intracellular release of the cytotoxic payload, either due to the endosomal reduction of the disulfide bond when cleavable linkers were used to graft the payload, or to a proteolytic degradation of the antibody part of the conjugate within the lysosome in the case of uncleavable thioether linker [42].

However, this activity was also associated with non-negligible side effects due to off target effects of the cytotoxic agent [43,44]. More recently, low toxic payloads such as the HDCA inhibitor ST7612AA1 have been grafted to monoclonal antibodies (cetuximab or trastuzumab), leading to better efficacy/toxicity ratios [45,46]. Along this line, our results demonstrate the feasibility of grafting high molecular weight payloads such as a 16-mer DNA mimic that are deprived of activity when they do not enter cells, but that induce significant cytotoxicity by a mechanism that could involve DNA topoisomerase I or other potential intracellular targets that still need to be identified. Due to the high similarity of these foldamers with B-DNA fragments, it is also tempting to surmise that intracellular delivery of our DNA mimic using this kind of trastuzumab conjugates may induce innate immune responses that could further potentiate the use of immune checkpoint inhibitors. This hypothesis is currently under investigation.

## 3. Materials and Methods

### 3.1. Chemical Synthesis and Characterization of a Modified DNA Mimic Foldamer

Chemical reagents were purchased from commercial suppliers (Sigma-Aldrich, Alfa-Aesar or TCI) and used without further purification. Low loading (LL) Wang resin (100–200 mesh, 1% DVB, manufacturer’s loading: 0.41 mmol g^−1^) was purchased from Novabiochem. Ghosez reagent (1-chloro-*N*,*N*,2-trimethyl-1 propenylamine) was purchased from Sigma–Aldrich. *N*,*N*-diisopropylethylamine (DIPEA) was distilled over CaH_2_ prior to use. Analytical grade organic solvents were used for solid phase synthesis. Anhydrous THF and CH_2_Cl_2_ for solid phase synthesis were dispensed from an *MBRAUN SPS-800* solvent purification system. RP-HPLC-quality acetonitrile and MilliQ water were used for RP-HPLC analyses and purification. RP-HPLC analyses were performed on a *Thermo Scientific Dionex UltiMate 3000* at 1.0 mL/min by using a Macherey–Nagel Nucleodur C18 HTec column (4 × 100 mm, 5 μm). The mobile phase was composed of 12.5 mM aqueous NH_4_OAc-NH_4_OH adjusted to pH 8.5 (solvent A) and CH_3_CN (solvent B). Monitoring was performed by UV detection at 214, 254 and 300 nm with a diode array detector. Semi-preparative purifications of oligomers were performed at 5 mL/min by using a Macherey–Nagel Nucleodur C18 HTEC column (10 × 125 mm, 5 μm). The mobile phase was the same as for the analytic system. Monitoring was performed by UV detection at 300 nm. ^1^H NMR spectra were recorded on an *Avance III HD 400 MHz Bruker BioSpin* spectrometer. Chemical shifts are reported in ppm relative to residual solvent signals of D_2_O (δ 4.79). Data processing was performed with Bruker TopSpin 4.0.6 software.

High-resolution electrospray mass spectra were recorded on a *Thermo Exactive orbitrap* instrument from the mass spectrometry service at the IECB (UMS3033 & US001).

### 3.2. Preparation of Quinoline Monomer and Solid Phase Synthesis of Oligomers **1** and **3**

The 8-Fmoc-amino-2-quinolinecarboxylic acid monomer (Fmoc-Q^Pho^) with a phosphonate group in position 4 protected as a di-*tert*-butyl ester was prepared by following the reported synthetic procedures [16]. Solid phase synthesis (SPS) of foldamer **1**, including Fmoc deprotection, acid chloride activation and coupling reactions (steps a–e, Scheme S1 of Supplementary Materials), were carried out by following the reported procedures [30]. To couple the linker at the foldamer’s *N*-terminus, H_2_N-(Q^Pho^)_16_-Wang resin (20 µmol) was suspended in anhydrous THF (1.25 mL) and 2,4,6-Collidine (9.0 equiv.) was added (step f, Scheme S1 of Supplementary Materials).

The diphenylthio-maleimido-caproic acid linker **2** (3.0 equiv) synthesized as previously described [25], and PPh_3_ (8.0 equiv.), were mixed in a plastic Eppendorf tube and dissolved with anhydrous CHCl_3_ (1.25 mL) before the addition of trichloroacetonitrile (TCAN, 9.0 equiv.). The reaction mixture was shaken in the Eppendorf tube before being quickly transferred to the pre-swollen resin. The reaction vessel was then placed under microwave irradiation (25 W, ramp to 50 °C over 5 min, then hold at 50 °C for 15 min). The resin was filtered off and washed with anhydrous THF (2 × 3 mL). The coupling step was repeated once using the same conditions and number of equivalents of coupling reagents. The resin was filtered off and washed with THF (3 × 3 mL) and DMF (2 × 3 mL). Resin cleavage and final TFA labile side chain-deprotection (step g, Scheme S1 of Supplementary Materials) were carried out as previously described [16] to yield **3** in 65% purified yield.

### 3.3. Bioconjugation, Purification and Characterization of the ADC **4**

Materials were obtained from commercial suppliers at the highest purity grade available and used without further purification. Disulfide bonds were reduced using a solution of Tris(2-carboxyethyl)phosphine (TCEP). The BBS conjugation buffer (Borate Buffered Saline) 1X was made at pH 8.0 with 25 mM NaCl, 1 mM EDTA and was titrated with NaOH 1M. The PBS buffer (Phosphate-Buffered Saline) 1X at pH 7.2 was used for the purification step of the ADC. The antibody trastuzumab was provided by le Centre Hospitalier Régional Universitaire (CHRU) de Tours. Antibody trastuzumab and trastuzumab-DNA mimic conjugate concentrations were determined by UV absorbance using NanoDrop-spectrophotometer (Thermo Fisher Scientific).

Conjugation of trastuzumab with diphenylthio-Mal-Cap-foldamer **3** (Figure 1C) was then performed as follows. To the purified trastuzumab (4.91 mg/mL, 33.16 µM, 10 × 200 µL, 148,068 Da) in BBS conjugation buffer was added TCEP (6 equiv, 1 mM in conjugation buffer, 10 × 39.79 µL) under argon and the reaction mixture was incubated at 37 °C for 75 min. Then, a solution of **3** (6 equiv, 1 mM in H_2_O MilliQ, 10 × 39.79 µL) was added and the reaction mixture was incubated for 2 h at 4 °C under stirring at 600 rpm. The crude conjugate was then purified by repeated ultrafiltration (GE Healthcare, 10,000 MWCO) into PBS Buffer (1× pH 7.2) and filtered through 0.22 µm filters. The purified ADC **4** was obtained at 13.37 µM (2.17 mg/mL). An average DAR of 3.0 was determined by LC-MS analysis according to our previously described method and the formula given below [17,25].

Characterization of the ADC **4** was performed by High Resolution Mass Spectrometry using an Acquity UPLC H-Class system hyphenated to a Vion IMS QTof mass spectrometer, both from Waters (Wilmslow, UK). Before MS analysis, 800 ng of sample was injected onto a BEH C4 2.1 × 30 mm, 1.7 µm column heated to 90°C. A desalting step was carried on with 95% solvent A (H_2_O + 0.1% formic acid) and 5% solvent B (acetonitrile + 0.1% formic acid) during 2 min at 0.5 mL/min, with the flow diverted to waste. Then, a 4 min gradient from 5% to 90% solvent B was applied with a 0.4 mL/min flow rate to elute the sample with the flow diverted to MS. MS data have been acquired using positive ionization mode with an ESI source over a 500 to 4000 *m/z* window with 1Hz scan. Voltage capillary was set to 2.5 kV, desolvation temperature and source temperature to 600 °C and 120 °C, respectively, and cone voltage 150 V. The results were processed using the UNIFI software version 1.9.4 and the MaxEnt1 algorithm for deconvolution. Deconvolution was carried out in the range 20–180 kDa, with results recorded for full antibody as well as fragments when present (comprising H, L, LH and LHH) as a result of antibody dissociation within the MS. The average DAR was calculated as an average of the percentage abundance of each present DAR species, with the quantities calculated by peak integration of the first glycosylation peak, following the general corresponding formula:

$$DAR_{average}=\frac{DAR_{LHHL}+\left(DAR_{LHH}+DAR_{L}\right)+2*DAR_{LH}}{3}$$

### 3.4. Cell Culture

MCF-7, T-47D, SK-BR-3 breast cancer cell lines and the SK-OV-3 ovarian cancer cell line were obtained from the American Type Culture Collection (ATCC). SK-OV-3 and MCF-7 cells were grown in Dulbecco’s Modified Eagle (DMEM) medium and SK-BR-3 and T-47D cells in RPMI-1640 medium supplemented with 10% fetal calf serum without antibiotics at 37 °C under a 5% CO_2_ humidified atmosphere. Cells were routinely checked for mycoplasma contamination using the MycoAlert^TM^ detection kit (Lonza, Basel, Switzerland).

### 3.5. Cell Growth Inhibition Assay

The effect of the different compounds on cell growth was evaluated using the sulforhodamine B (SRB) assay, as previously described [47]. Briefly, 3000–5000 cells/well were seeded in 96-well plates. After 24 h, cells were incubated with indicated concentrations of each compound for 72 h. Then, the medium was removed and cells were fixed with a trichloroacetic acid solution (10% final concentration) and stained with 0.4% SRB solution in 1% acetic acid for 30 min. Cells were washed three times with 1% acetic acid and SRB was dissolved in 10 mmol/L Tris-HCl solution by gentle shaking. Absorbance at 560 nm was then measured using a PHERAstar FS plate reader (BMG Labtech, Champigny s/Marne, France). Percent growth was calculated as compared to untreated cells and plotted as a function of concentrations. Results are the mean ± sd of three independent experiments.

### 3.6. Analysis of Antibody-Drug Conjugate Internalization by Immunofluorescence

Internalization of the trastuzumab-DNA mimic ADC in breast and ovarian cancer cells was studied by immunofluorescence. Briefly, 3 × 10^4^ cells were seeded on coverslips in 24-well plates. Two days later, exponentially growing cells were incubated for 2 h without or with 15 µg/mL of traztuzumab alone or of the ADC, at 4 °C or at 37 °C. Then, supernatants were removed and cells were washed twice with PBS-Tween 1% and once with PBS. Cells were then fixed by a 40 min incubation in formalin (3.7% formaldehyde in PBS) and permeabilized with 0.5% Triton X-100/PBS for 15 min. The cells were washed twice with PBS and incubated in PBS/BSA (2%) for 40 min. They were further incubated with a goat FITC-conjugated anti-human IgG antibody (F9512, Sigma-Aldrich, 1:200 dilution) for 90 min, washed three times with PBS-Tween 1% and three times with PBS and mounted with Everbrithe ^®^ (Biotium, San Francisco, CA, USA) with DAPI, and were visualized using an epifluorescence Zeiss Imager 2 (Zeiss, Germany).

### 3.7. Flow Cytometry Experiments

The binding of the ADC and trastuzumab to HER2 was assessed using a fluorescence-activated cell sorter (Quanta apparatus, Beckman Coulter). For each cell line, one million cells were pelleted, washed with PBS/1% BSA and incubated with 10 µg/mL of trastuzumab or of the ADC on ice for 1 h. Then, cells were washed with PBS/1% BSA and incubated with a goat FITC-conjugated anti-human IgG antibody (F9512, Sigma-Aldrich, 1:200 dilution) on ice for 1 h. The irrelevant 13R4 antibody targeting beta-galactosidase was used as a negative control and incubation of cells with the secondary antibody was only used for background measurements. The gating strategy that was used for FACS analyses is presented in Figure S6.

## 4. Conclusions

The ADCs that are approved or in clinical development for cancer treatment are almost exclusively based on the use of highly cytotoxic payloads. To our knowledge, nucleic acid mimics have never been used as payloads, though they are known to interfere with the function of DNA interacting enzymes and/or modulate the expression of genes involved in cancer cell growth. Our study provides the first experimental evidences that it is possible to conjugate high molecular weight oligoamide-based DNA mimics onto monoclonal antibodies with a controlled drug–antibody ratio, and that the resulting ADC binds to its extracellular target before its internalization into cells. Our results further extend the potential of using the ADC strategy to deliver other categories of payloads that are not necessarily highly cytotoxic but would rather be used to potentiate the action of other drugs that are administered in combination, including conventional chemotherapies.

## Figures and Tables

**Table 1:** Mean fluorescence intensity (Gmean) values following cell incubation with the ADC, trastuzumab or the irrelevant 13R4 antibody as measured by FACS. Values are the mean of fluorescence intensities obtained from two independent experiments (indicated in parentheses).

	13R4	TZ	ADC
**MCF-7**	**0.45** (0.6, 0.3)	**13.1** (14.1, 12.2)	**6.7** (7.2, 6.2)
**T-47D**	**0.4** (0.3, 0.5)	**85.0** (65.9, 104.0)	**83.6** (65.1, 102.0)
**SK-BR-3**	**0.4** (0.3, 0.45)	**88.0** (89.0, 87.0)	**84.6** (86.1, 83.0)
**SK-OV-3**	**0.5** (0.5, 0.5)	**223.5** (222.0, 225.0)	**180.0** (177.0, 183.0)

## Data Availability

Data is contained within the article and supplementary material.

## Supplementary Materials

The following are available online at https://www.mdpi.com/article/10.3390/ph14070624/s1, Scheme S1: Solid Phase Synthesis of diphenylthio-Mal-Cap-foldamer 3 (See experimental procedures for details), Figure S1: ^1^H NMR spectra (400 MHz) in H_2_O/D_2_O 9:1 (vol/vol), 50 mM NH_4_HCO_3_ at 298 K before (bottom) and after (top) the coupling reaction with the diphenylthio-maleimido-caproic acid linker 2, Figure S2: Analytical RP-HPLC profile of pure diphenylthio-Mal-Cap-foldamer 3, Figure S3: Multicharged species observed by ESI HRMS analysis (anionic mode) of pure dithiophenyl-Mal-Cap-foldamer 3, Figure S4: Internalization of the ADC or trastuzumab in MCF-7 and T-47D cells, Figure S5: MS analysis of ADC Trastuzumab-Mal-Cap-foldamer 4, Figure S6: Gating for FACS analysis, Table S1: Average drug-to-antibody ratio (DAR) calculation.
