# Supplementary material for: Internalization of Foldamer-Based DNA Mimics through a Site-Specific Antibody Conjugate to Target HER2-Positive Cancer Cells
Source: Pharmaceuticals (Basel). 2021 Jun 28;14(7):624. doi: 10.3390/ph14070624 (PMC8308903; doi:10.3390/ph14070624)
Supplement: Supplementary file 1 [file pharmaceuticals-14-00624-s001.zip › pharmaceuticals-1264476-supplementary.pdf]

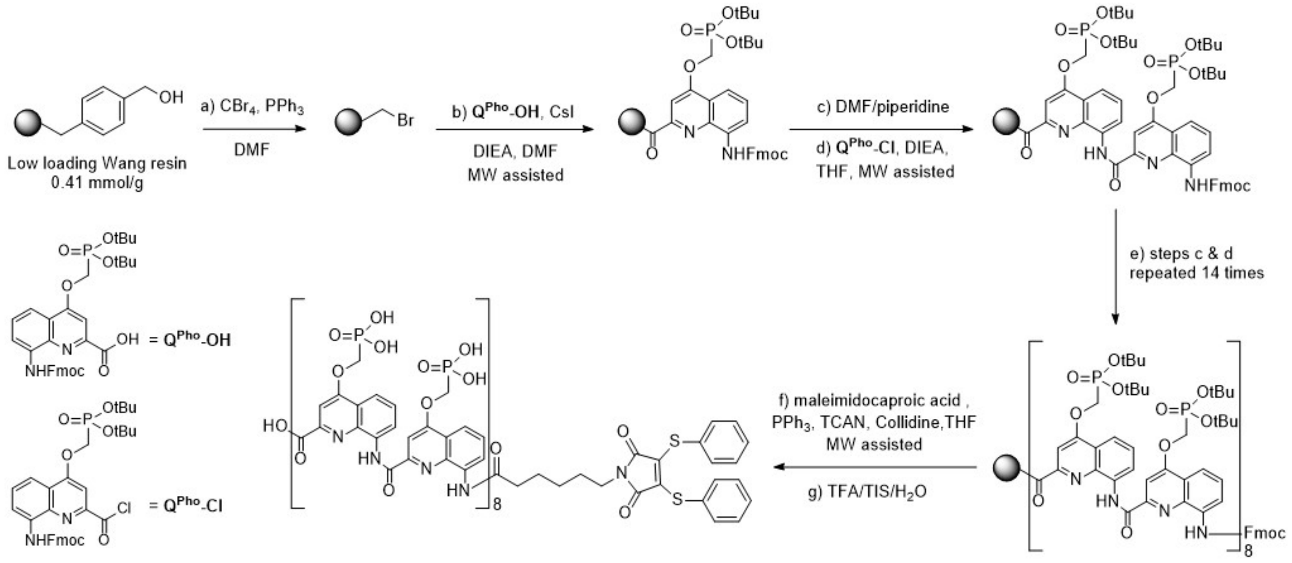

Scheme S1: Solid Phase Synthesis of diphenylthio-Mal-Cap-foldamer **3** (See experimental procedures for details).

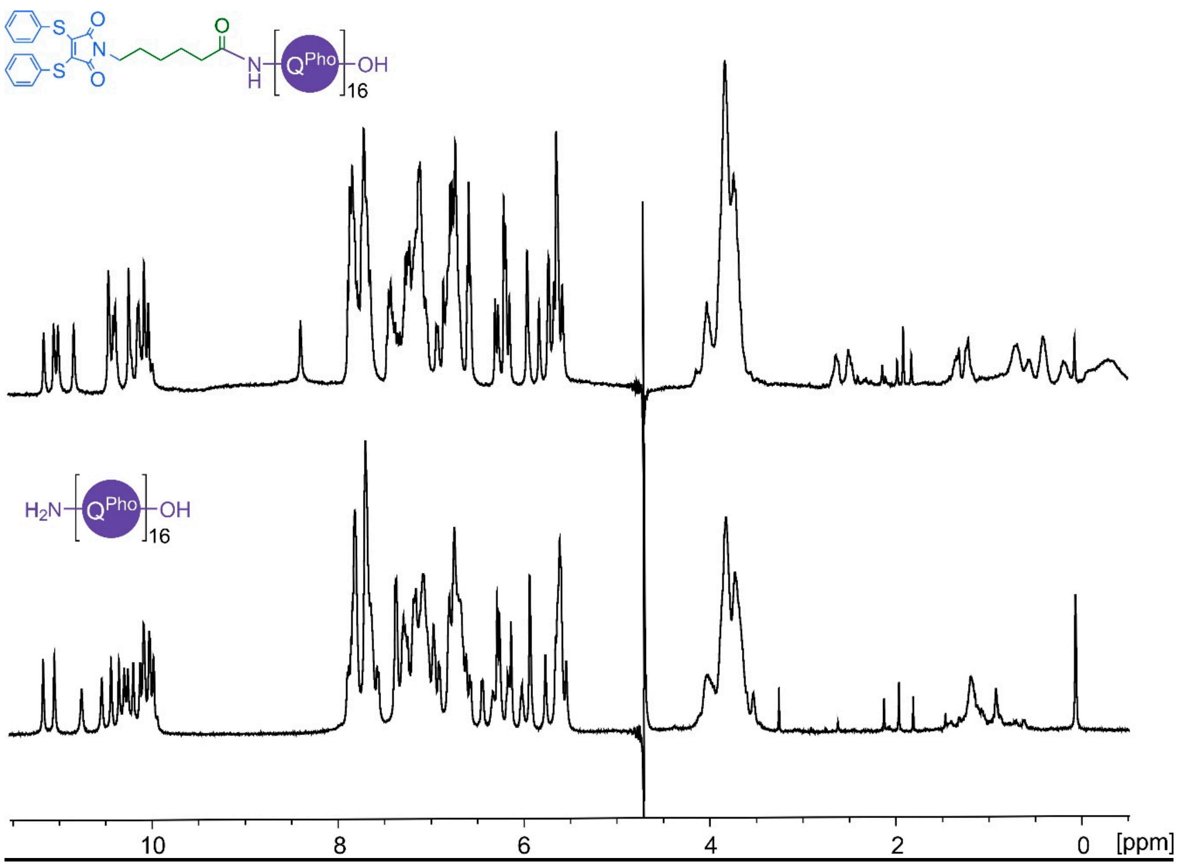

Figure S1. ^{1}H NMR spectra (400 MHz) in H_2O/D_2O 9:1 (vol/vol), 50 mM NH_4HCO_3 at 298 K before (bottom) and after (top) the coupling reaction with the diphenylthio-maleimido-caproic acid linker **2**. The additional carboxamide signal at 8.38 ppm in the top NMR spectrum indicates the successful coupling reaction.

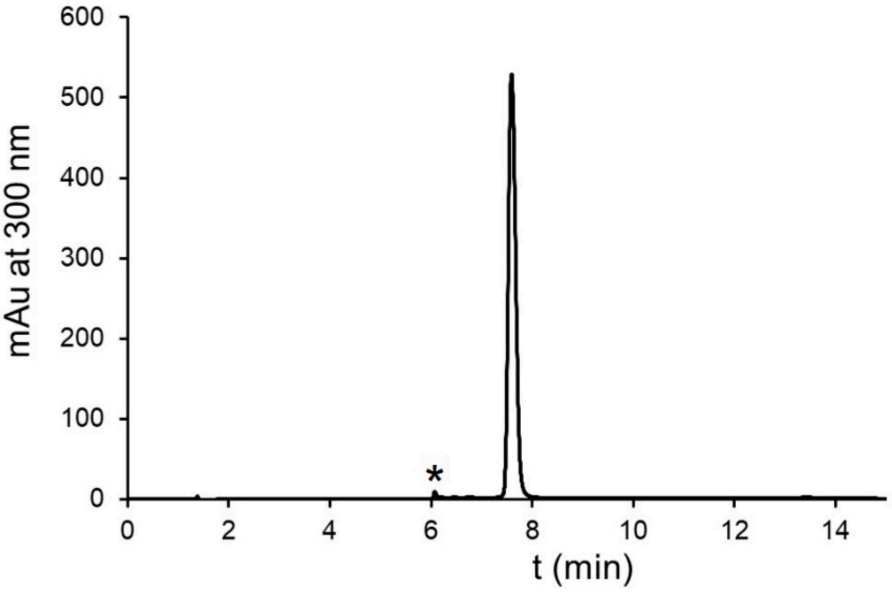

Figure S2. Analytical RP-HPLC profile of pure diphenylthio-Mal-Cap-foldamer **3**. Elution conditions: Nucleodur C18 HTec column (4 x 100 mm, 5 µm); UV detection at λ = 300 nm; gradient of eluents A and B, where A was NH_4OAc-NH_4OH adjusted to pH 8.5 and B was CH_3CN; gradient program from 100% A, 0% B to 50% A, 50% B over 10 min. A small impurity from the solvent is denoted with an asterisk.

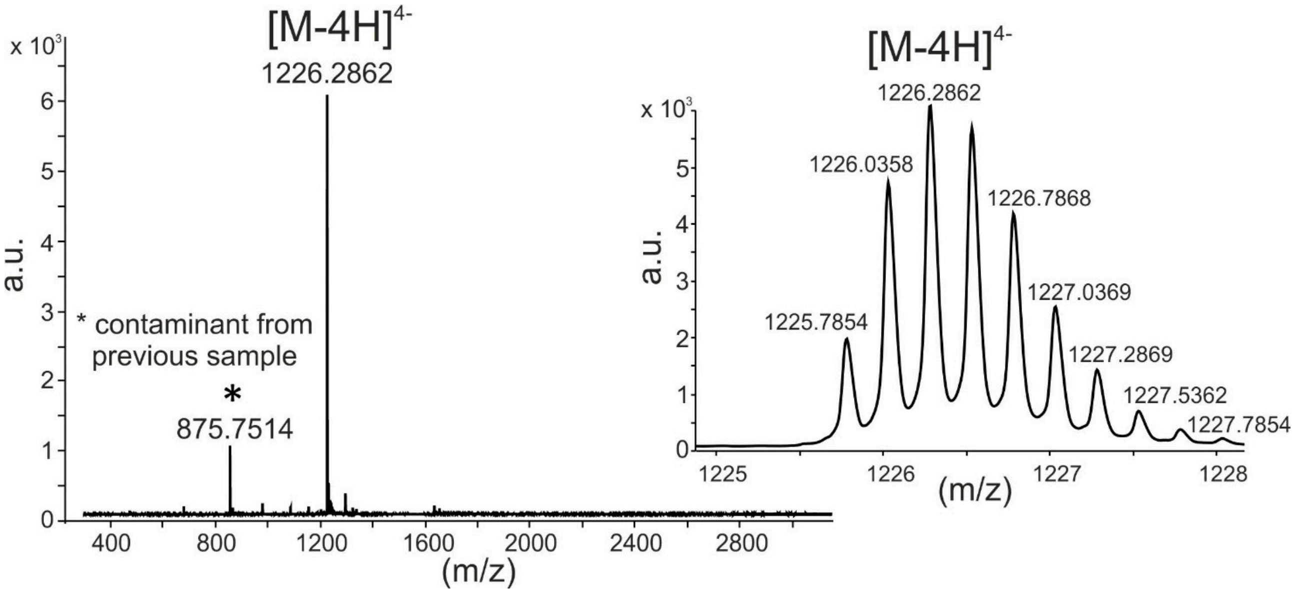

Figure S3. Multicharged species observed by ESI HRMS analysis (anionic mode) of pure dithiophenyl-Mal-Cap-foldamer **3**.

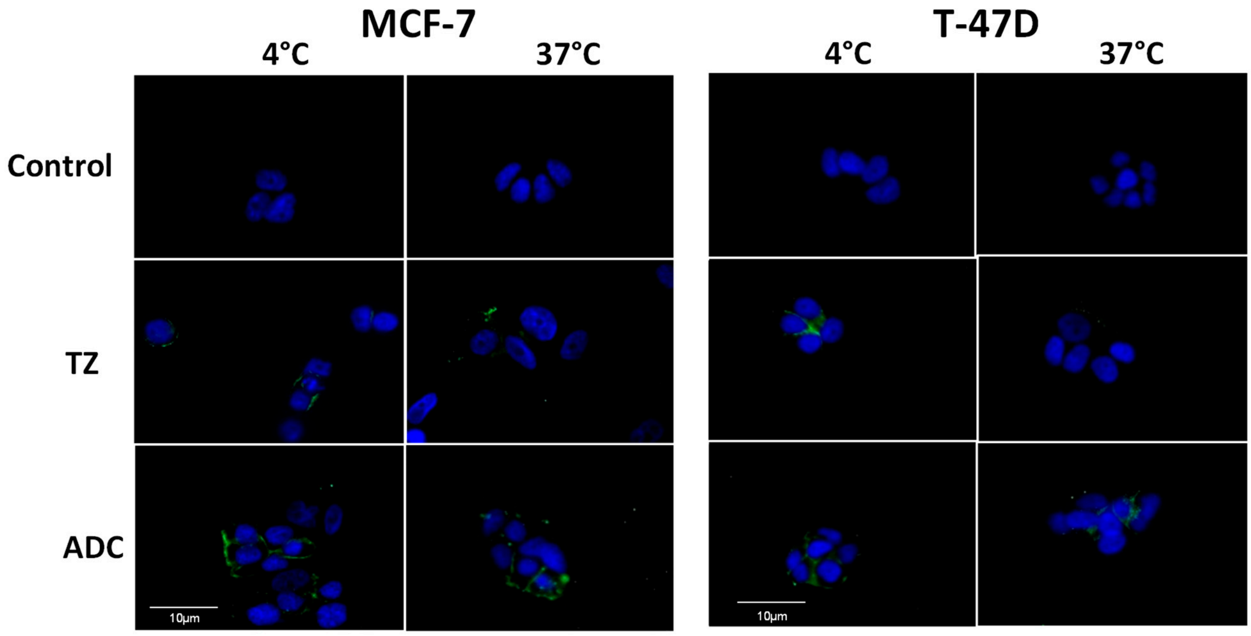

Figure S4. Internalization of the ADC or trastuzumab in MCF-7 and T-47D cells at 4°C and 37°C as described in figure 4.

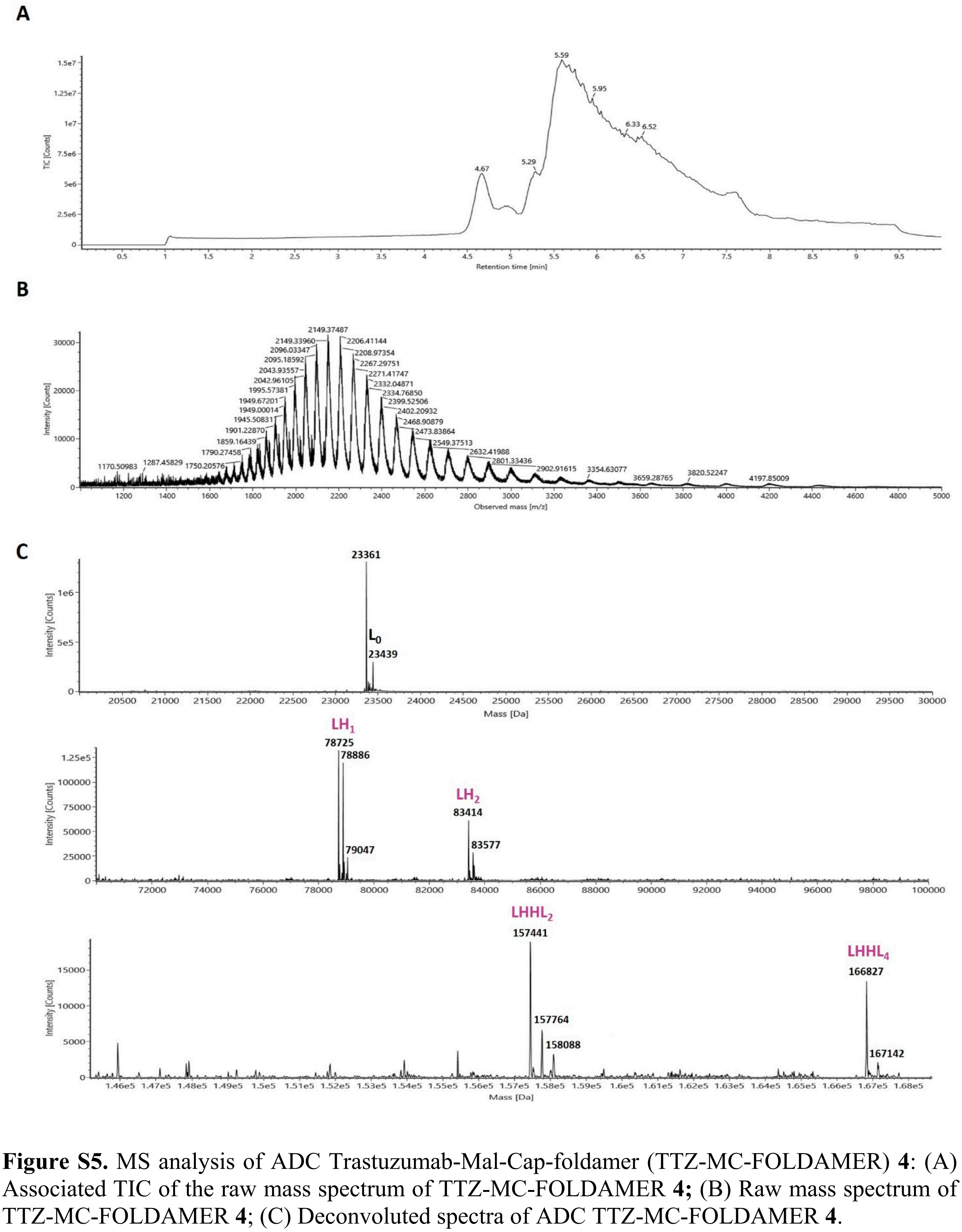

Figure S5. MS analysis of ADC Trastuzumab-Mal-Cap-foldamer (TTZ-MC-FOLDAMER) **4**: (A) Associated TIC of the raw mass spectrum of TTZ-MC-FOLDAMER **4;** (B) Raw mass spectrum of TTZ-MC-FOLDAMER **4**; (C) Deconvoluted spectra of ADC TTZ-MC-FOLDAMER **4**.

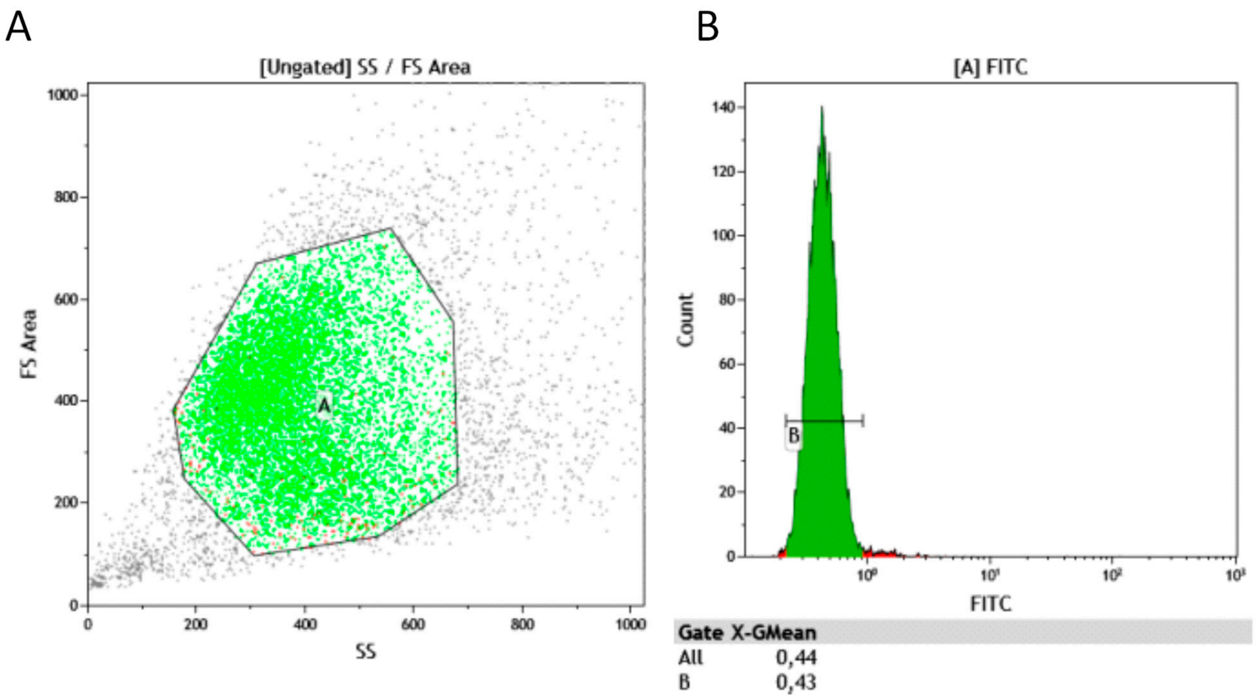

Figure S6. Gating for FACS analysis. (A) Cell population is selected using forward scatter (FS) and side scatter (SS), according to their size and granulometry. Cell debris and doublets are eliminated. (B) FITC staining analysis was first performed on unlabelled cells in order to define the gate of FITC negative cells for each cell line.

Table S1. Average drug-to-antibody ratio (DAR) calculation.

Species[1]	DAR species[1]	Area intensity	Proportion per species (%)	Relative DAR	Average DAR per species
LHHL	DAR 2	525912	43	0.86	3.14
	DAR 4	685610	57	2.28	
LH	DAR 1	4969662	57	0.57	1.43
	DAR 2	3607535	43	0.86	
Average DAR[2]	**3.00**				

[1] species not mentioned were not observed or not relevant.

[2] calculated according to the mentioned species with the corresponding formula:

$$DAR_{average} = \frac{DAR_{LHHL}+2*DAR_{LH}}{2}$$
